# Transient aplastic crisis triggered by parvovirus B19 in a family with hereditary spherocytosis

**DOI:** 10.1016/j.idcr.2020.e00802

**Published:** 2020-05-11

**Authors:** Nicolas Cilla, Léa Domitien, Neila Arrada, Delphine Chiffre, Perrine Mahe, Laure Vincent, Patricia Aguilar-Martinez, Vincent Foulongne

**Affiliations:** a Department of Clinical Hematology, Montpellier University Hospital, 34295, Montpellier, France; b Division of Hematologic Diseases, Department of Pediatrics, Montpellier University Hospital, University Montpellier, Montpellier, France; c Pathogenesis and Control of Chronic Infections, University of Montpellier, INSERM, EFS, Montpellier University Medical Centre, 34090, Montpellier, France; d Department of Biological Hematology, Montpellier University Medical Centre & University of Montpellier, Montpellier, France; e Reference Center on Rare Red Cell Disorders, Montpellier University Medical Centre, Montpellier, France

**Keywords:** Parvovirus B19, Hereditary spherocytosis, Erythroblastopenia

## Abstract

Acute parvovirus B19 infection may lead to erythroblastopenia crisis in patients with underlying red blood cells disorders. We report herein an uncommon concomitant transient aplastic crisis in a mother and her daughter, both affected by hereditary spherocytosis. The diagnosis was confirmed by the detection of a very high parvovirus B19 DNA load in both the mother’s and daughter’s sera, associated with the presence of parvovirus B19 specific immunoglobulin-M antibodies. This rapid etiologic diagnosis allowed to save bone marrow sampling, although blood transfusion was required regarding the severe anemia associated with pancytopenia. Our observation illustrates first line parvovirus B19 hypothesis in the context of transient aplastic crisis and that contagiousness in household contacts should be considered in family with a medical history of red blood cell pathology.

## Introduction

Parvovirus B19 is a human pathogenic member of the *Parvoviridae* family and is the etiologic agent of the erythema infectiosum, a mild rash illness in childhood [1]. In adults, parvovirus B19 infection can be associated with post infectious arthralgia. However, the most significant manifestations associated with parvovirus B19 infections are related to the original tropism of the virus for the erythroid progenitor cell line [1,2]. It can therefore trigger pure red cell aplasia and chronic anemia in immunocompromised hosts and is associated, in immature susceptible fetus, with hydrops foetalis, congenital anemia or fetal death [1]. Likewise, parvovirus B19 infection occurring in patients with underlying hemolytic disorders may cause a transient aplastic crisis with a marked drop in hemoglobin. Parvovirus B19-induced aplastic crisis can be observed in patients with decreased red blood cells (RBC) production observed in iron deficiency anemia or thalassemias for example [3], as well as in patients with increased RBC loss. This includes chronic hemolytic anemias, such as sickle cell disease, glucose-6-phosphate deshydrogenase (G6PD) deficiency, hereditary stomatocytosis, or, as illustrated through the present description, hereditary spherocytosis (HS) [4,5].

## Case reports

### The daughter

A 12-year-old girl was admitted to the emergency unit of Montpellier University Hospital for nausea, vomiting and headache. She was febrile (39 °C) with an associated tachycardia (144 b/min) and normal blood pressure (121/55 mmHg). Abdominal examination was normal, with no pain and absence of hepatosplenomegaly. Neither rash nor signs of dehydration were observed. She had slight conjunctival icterus. She was breathing normally without dyspnea. No neurological symptoms were observed. Blood tests revealed a significant drop of hemoglobin level (6,4 g/dL) concurrent with a mild thrombocytopenia (140,000 /μL) and leucopenia (2800 /μL), with 102,000 /μL reticulocytes. Examination of the blood smear revealed abnormally shaped RBC, including spherocytes, and significant poikilocytosis. The other laboratory findings were a low haptoglobin level (<10 mg/dL), an increased C-reactive protein (41.5 mg/L), and a high level of serum ferritin (8575 ng/dL) and LDH (405 IU/L). A diagnosis of non-regenerative hemolytic anemia was proposed and she was hospitalized in the pediatric department. Serological investigation showed parvovirus B19 immunoglobulin-M (IgM) antibodies and a specific parvovirus B19 PCR on the same serum yielded a strong positive signal with an unusual early cycle threshold (Ct) (i.e. Ct<5), suggesting a very high viral load. During hospitalization, hemoglobin levels dropped to a minimum of 4,8 g/dL. The anemia was corrected with two blood transfusions on days 2 and 6 post admission. The girl was discharged home after 7 days with a final diagnosis of transient aplastic crisis following parvovirus B19 acute infection in a likely context of hereditary spherocytosis. During the course of her hospitalization, her mother was admitted for similar symptoms in the adult hematology department.

### The mother

**Fig. 1:**
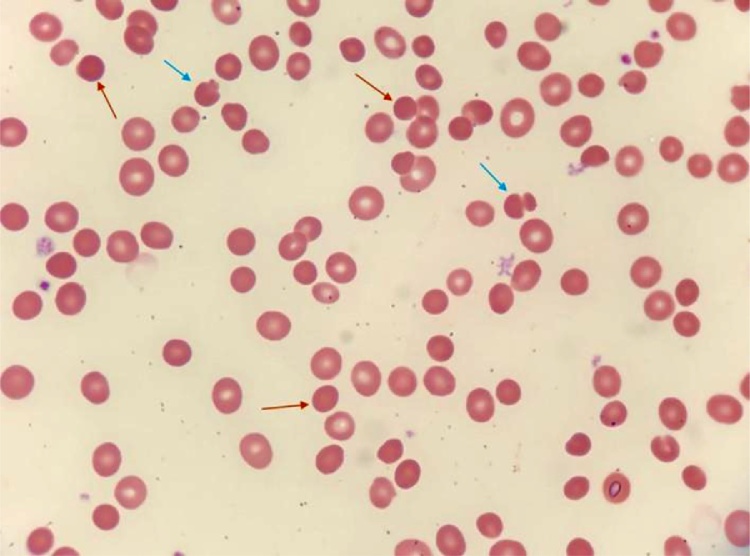
May-Grunwald Giemsa stained blood smear of the mother in the acute phase. Presence of numerous spherocytes (red arrows) and some mushroom cells (blue arrows). (For interpretation of the references to colour in this figure legend, the reader is referred to the web version of this article).

Four days after her daughter’s admission, the 34 year-old mother was admitted for intense weakness, palpitations and dyspnea following efforts. A discrete splenomegaly protruding 1 cm beyond the mid-clavicular line was detected during abdominal examination. Blood tests revealed a similar pancytopenia with macrocytic non-regenerative anemia (hemoglobin 5,8 g/dL, MCV 101 fL, low reticulocyte count at 27,000 /μL; 119,000 /μL platelets and 3500 /μL WBC with neutropenia 3500 /μL and lymphopenia 780 /μL). On the blood smears, up to 40 % of spherocytes were observed with many mushroom red cells (Fig. 1), which are found in HS due to protein band 3 deficiency. The other laboratory findings were a discrete hyponatremia (130 mmol/L), a low haptoglobin level (<10 mg/dL) and high serum ferritin levels (8394 ng/dL) and LDH (410 IU/L). The G6PD activity was normal (11.7 IU/g Hb). The direct antiglobulin test was negative and there was no associated vitamin deficiency. Serological investigations confirmed the presence of parvovirus B19 IgM antibodies. A specific parvovirus B19 PCR signal as intense as that observed for her daughter was detected. She received as well blood transfusions and was discharged home 2 days later. A second set of transfusion was necessary 8 days later regarding the persistence of anemia (Hb 5,3 g/dL), until a 8,2 g/dL hemoglobin level was achieved 3 days later. Final retained diagnosis was pancytopenia following acute parvovirus B19 infection in a context of hereditary spherocytosis, further confirmed by positive Eosin-5′-Maleimide (EMA) test. This test demonstrated an abnormal binding of EMA to the erythrocyte membrane with a rate of change from controls of 24.7 %.

## Discussion

Parvovirus B19 infection is very common and ubiquitous in human. The prevalence of IgG antibodies against B19 ranges from 15 to 60 % in adolescents and up to 80 % in adults [6]. The spectrum of diseases linked to parvovirus B19 varies with the immune and hematologic status of the host. Erythema infectiosum or fifth disease is the most common manifestation in immunocompetent children whereas in adults, infection is often associated to arthritis or arthralgia underlying a likely immunomediated mechanism [1]. In immunocompromised patients, B19 infection can induce chronic pure red cell aplasia and during pregnancy, B19 infection can impair the immature fetal erythropoiesis leading to hydrops foetalis and congenital anemia.

When parvovirus B19 infection occurs in patients with underlying red cells disorders, the cytopathic effect of the virus on red blood cell progenitors majors the red cell aplasia leading to a transient aplastic crisis. Any conditions that impair the production or accelerate the destruction of erythrocytes are at high risk for aplastic crisis following B19 acute infection. The most frequent conditions include: sickle cell disease, hemoglobin C disease, alpha or beta-thalassemias, red cell enzyme deficiencies (G6PD, pyruvate kinase), autoimmune hemolytic anemia, paroxysmal nocturnal hemoglobinuria, hereditary elliptocytosis as well as hereditary spherocytosis [3,4]. In our observation both patients, mother and daughter, were affected by hereditary spherocytosis. During the course of their hospitalization, such underlying condition was raised, since the mother was aware of family cases, reporting three uncles and a brother previously diagnosed with hereditary spherocytosis. This diagnosis had not been previously investigated for this woman and her daughter, but was precipitated through the episode of acute B19 infection. In predisposed individuals 70–80% of aplastic crisis are caused by B19 infection [3], but the occurrence of simultaneous cases within a family remains a rare event, previously described in only few reports [[8], [9], [10]]. Interestingly, in most published cases, the aplastic crisis was activated by the parvovirus B19 infection and revealed the underlying RBC anomalies. Both, mother and daughter have been referred to the RBC pathology reference center of our hospital for medical advice regarding their congenital disorder.

Hereditary spherocytosis is the commonest (1/2000) cause of inherited chronic hemolysis in caucasians. The inheritance is mainly autosomal dominant but can also be autosomal recessive, as well as sporadic [11]. This red cell membrane disorder is due to mutations of genes encoding proteins constitutive of the ankyrin-complex with, as a consequence, a decreased attachment of the spectrin-based membrane cytoskeleton to the lipid bilayer. This results in spherical red cells with reduced deformability that will be sequestered in the spleen. Blood smear examination shows the presence of variable numbers of spherocytes, and the diagnosis can be confirmed through osmotic fragility tests or by the EMA tests. Further confirmation can include Ektacytometry and in difficult cases, characterization of the molecular defect can be proposed. As observed for the two presented patients, resulting hemolysis is usually well-compensated, except when an additional event occurs. In severe cases, treatment relies on splenectomy, however its benefit remains to be discussed regarding each specific cases.

Parvovirus B19 is a contagious virus through an airborne transmission route and could lead to small outbreaks, mainly in community of young children with an attack rate close to 50 % among susceptible seronegative contacts [1]. In our observation, the young girl of school age has probably first contracted the infection with a subsequent household transmission to her mother. This illustrates that prevention of such infection remains very difficult regarding the contagiousness of the virus and the absence of warning infectious symptoms prior to the triggering of the aplastic crisis. There is unfortunately no currently available parvovirus B19 vaccine, although it could provide substantial benefit to prevent aplastic crisis in such patients with underlying RBC diseases.

In the present cases, investigation for the parvovirus B19 etiology immediately followed the evidence of severe anemia, thus allowing a rapid diagnosis and the save of more invasive bone marrow investigations. The diagnosis of acute parvovirus B19 infection is easy with the detection of IgM antibodies in early infection since 85 % of patient exhibit IgM in acute phase [1,6]. These antibodies are expected to act as neutralization antibodies leading to virus clearance. In our observation, the viral load was very high, up to more than 8 Log copies/mL, reflecting an intense viral replication suggested as well by the deep RBC decrease. High viral load as a marker of acute primary infection is commonly described in many studies [7].

In acute infections of patients with red cell disorders, although the clinical presentation is frequently severe regarding the drop in the hemoglobin level and the associated pancytopenia, the outcome is most of the time favorable when anemia is corrected. Standard care procedures rely on blood transfusions when necessary, whereas infusion of immunoglobulins may be proposed for immunocompromised patients [5]. For both described patients, a favorable outcome was observed after achieving satisfactory hemoglobin levels through blood transfusion. Complete recovery of normal blood cell count was obtained two months later. Seroconversion with the raise of IgG antibodies was completed as well. However a remaining low level viremia was still detected confirming the slow parvovirus B19 clearance frequently observed following acute infection [12].

Our observation describes an uncommon situation of intrafamilial aplastic crisis triggered by parvovirus B19 that have revealed the underlying hereditary spherocytosis. It illustrates the central place of parvovirus B19 diagnosis supported by both, serology and virus DNA detection.

## Ethic statements

Written inform consents were obtained from patients described in this manuscript.

## Author statement

N. Cilla and V. Foulongne co-wrote the article.

L. Vincent and N. Cilla took care of the mother in the clinical haematology department and collected the clinical information needed to write the case.

L. Domitien and P. Mahe took care of the daughter in the paediatric department and collected the clinical information needed to write the case.

N. Arrada collected the biological data

P. Aguilar-Martinez was approached for his expertise in erythrocytic pathologies.

V. Foulonge heads the virology laboratory where the parvovirus B19 PCRs were performed and actively participated in the proofreading of this article.

## Declaration of Competing Interest

No conflict of interest to declare

## References

[1] Qiu J., Söderlund-Venermo M., Young N.S. (2017). Human parvoviruses. Clin Microbiol Rev.

[2] Brown K.E., Anderson S.M., Young N.S. (1993). Erythrocyte P antigen: cellular receptor for B19 parvovirus. Science.

[3] Brown K.E. (2000). Haematological consequences of parvovirus B19 infection. Clin Haematol.

[4] Pattison J.R., Jones S.E., Hogson J., Davis L.R., White J.M., Stroud C.E. (1981). Parvovirus infections and hypoplastic crisis in sickle-cell anaemia. Lancet.

[5] Harris J.W. (1992). Parvovirus B19 for the hematologist. Am J Hematol.

[6] Maple P.A.C., Hedman L., Dhanilall P., Kantola K., Nurmi V., Söderlund-Venermo M. (2014). Identification of past and recent parvovirus B19 infection in immunocompetent individuals by quantitative PCR and enzyme immunoassays: a dual-laboratory study. J Clin Microbiol.

[7] Brown K.E. (2004). Detection and quantitation of parvovirus B19. J Clin Virol.

[8] Granel B., Serratrice J., Rey J., David M., Pache X., Bernit E. (2001). Erythroblastopénie aiguë transitoire intrafamilliale et sphérocytose héréditaire: responsabilité du parvovirus B19. Rev Med Interne.

[9] Sato T., Ueda D., Sakota S., Haseyama K., Chiba S. (1999). Pancytopenia with hemophagocytosis secondary to parvovirus b19 infection in a family with hereditary spherocytosis. Pediatr Int (Roma).

[10] Skinninder L.F., McSheffrey B.J., Sheridan D., Deener H. (1998). Congenital spherocytic hemolytic anemia in a family presneting with transient red cell aplasia from parvovirus B19 infection. Am J Hematol.

[11] Perrotta S., Gallagher P.G., Mohandas N. (2008). Hereditary spherocytosis. Lancet.

[12] Lindblom A., Isa A., Norbeck O., Wolf S., Johansson B., Broliden K. (2005). Slow clearance of human parvovirus B19 following acute infection. Clin Inf Dis..

